# Author Correction: TLR agonists polarize interferon responses in conjunction with dendritic cell vaccination in malignant glioma: a randomized phase II Trial

**DOI:** 10.1038/s41467-024-48995-7

**Published:** 2024-06-05

**Authors:** Richard G. Everson, Willy Hugo, Lu Sun, Joseph Antonios, Alexander Lee, Lizhong Ding, Melissa Bu, Sara Khattab, Carolina Chavez, Emma Billingslea-Yoon, Andres Salazar, Benjamin M. Ellingson, Timothy F. Cloughesy, Linda M. Liau, Robert M. Prins

**Affiliations:** 1 Department of Neurosurgery, David Geffen School of Medicine at UCLA, University of California Los Angeles, Los Angeles, CA 90095 USA; 2 Jonsson Comprehensive Cancer Center, David Geffen School of Medicine at UCLA, University of California Los Angeles, Los Angeles, CA 90095 USA; 3 Department of Medicine, Division of Dermatology, David Geffen School of Medicine at UCLA, University of California Los Angeles, Los Angeles, CA 90095 USA; 4 Parker Institute for Cancer Immunotherapy, David Geffen School of Medicine at UCLA, University of California Los Angeles, Los Angeles, CA 90095 USA; 5 Department of Molecular and Medical Pharmacology, David Geffen School of Medicine at UCLA, University of California Los Angeles, Los Angeles, CA 90095 USA; 6 Oncovir, Inc., Winchester, VA USA; 7 Department of Radiological Sciences, David Geffen School of Medicine at UCLA, University of California Los Angeles, Los Angeles, CA 90095 USA; 8 Department of Neurology/Neuro-Oncology, David Geffen School of Medicine at UCLA, University of California Los Angeles, Los Angeles, CA 90095 USA

**Keywords:** Immunotherapy, CNS cancer

Correction to: *Nature Communications* 10.1038/s41467-024-48073-y, published online 08 May 2024

In this article the author name Sara Khattab was incorrectly written as Sarah Khattab. The original article has been corrected.

