# Supplementary material for: TLR agonists polarize interferon responses in conjunction with dendritic cell vaccination in malignant glioma: a randomized phase II Trial
Source: Nat Commun. 2024 May 8;15:3882. doi: 10.1038/s41467-024-48073-y (PMC11078958; doi:10.1038/s41467-024-48073-y)
Supplement: Supplementary file 1 — Supplementary Information [file 41467_2024_48073_MOESM1_ESM.pdf]

Supplementary Figure 1. CONSORT diagram of clinical trial enrollment.

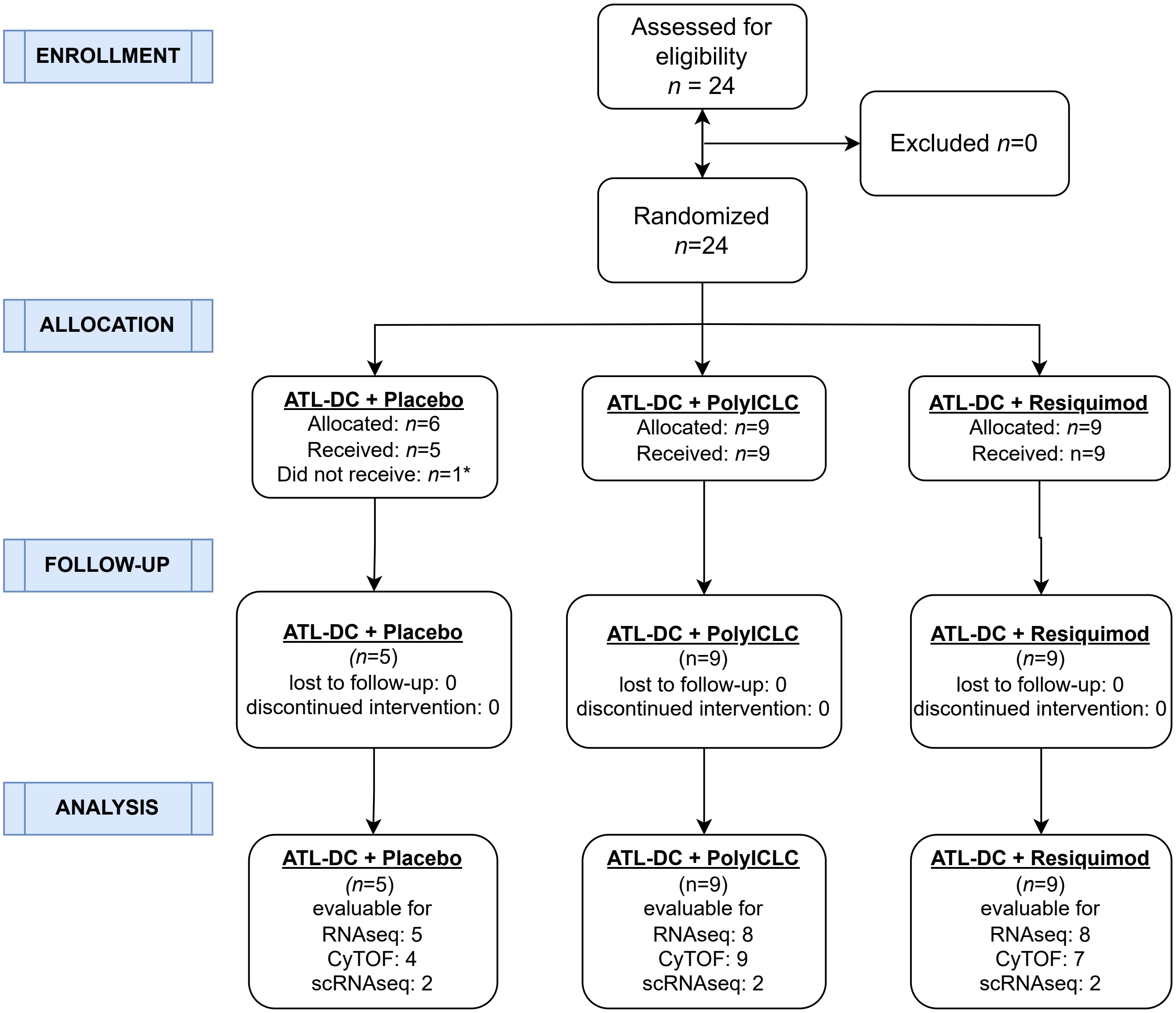

*due to early progression during vaccine manufacture

Supplementary Figure 1. CONSORT diagram of clinical trial enrollment.

Supplementary Figure 2

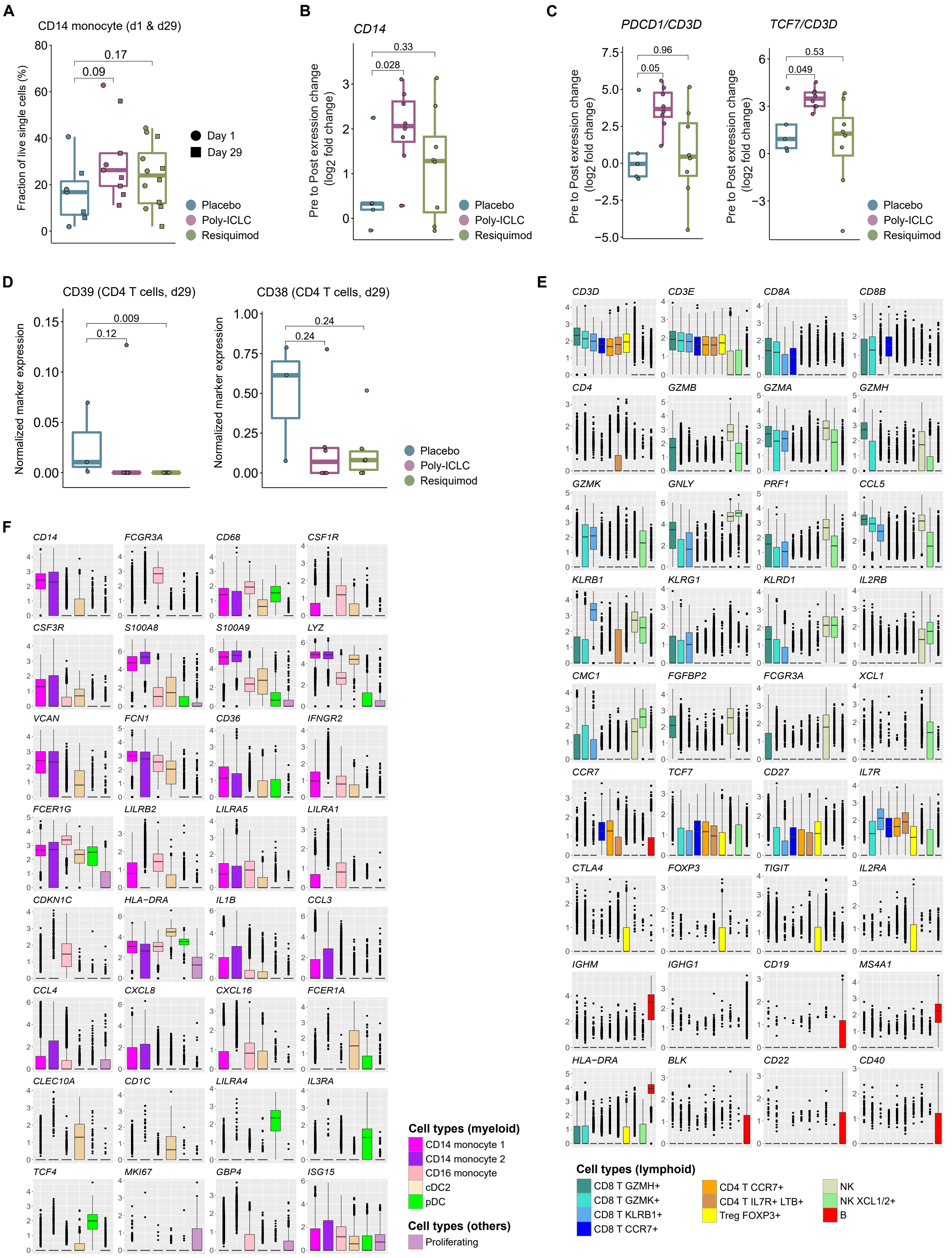

Supplementary Figure 2. CyTOF and single cell transcriptomics of patient PBMCs before and after ATL-DC vaccine with or without adjuvant TLR agonist.
A, Comparison of CD14+ monocyte fraction in post-treatment PBMCs of patients from indicated treatment groups. P values, two-sided Wilcoxon rank sum test.
B, Differential gene expression (pre vs. post-treatment fold change, in $\log_2$) of *CD14* transcript across treatment groups (P values, two-sided Welch t test).
C, Differential gene expression (pre vs. post-treatment fold change, in $\log_2$) of *PDCD1* transcript across treatment groups (P values, two-sided Welch t test) after adjusting for the change in *CD3D* transcript expression in the same sample pair. The values approximate the changes of *PDCD1* transcript per T cell.
D, Normalized expression of indicated markers in CD4 T cell populations within the PBMC samples of patients from indicated treatment groups. P values, two-sided Wilcoxon rank sum test.
E, F, Boxplots showing marker gene expressions in lymphoid cell populations (**E**) or myeloid and proliferative cell populations (**F**).

The number of sample pairs analyzed by CyTOF in **A** and **D** are: ATL-DC+placebo, 4 pairs; ATL-DC+poly-ICLC, 9 pairs; ATL-DC+resiquimod, 7 pairs.
The number of sample pairs analyzed by RNAseq in panels B and C are: ATL-DC +placebo, 5 pairs; ATL-DC+poly-ICLC, 8 pairs; ATL-DC+resiquimod, 8 pairs.

The rectangular box in each boxplot represents the interquartile range (IQR), spanning from the first quartile (25th percentile, bottom of box) to the third quartile (75th percentile top of box). Inside the box, the median (50th percentile) is marked. The whiskers (shown as lines extending from the box) extend to the largest and smallest non-outlier values within 1.5 times the IQR, while outliers lie beyond the whiskers.

Supplementary Figure 3

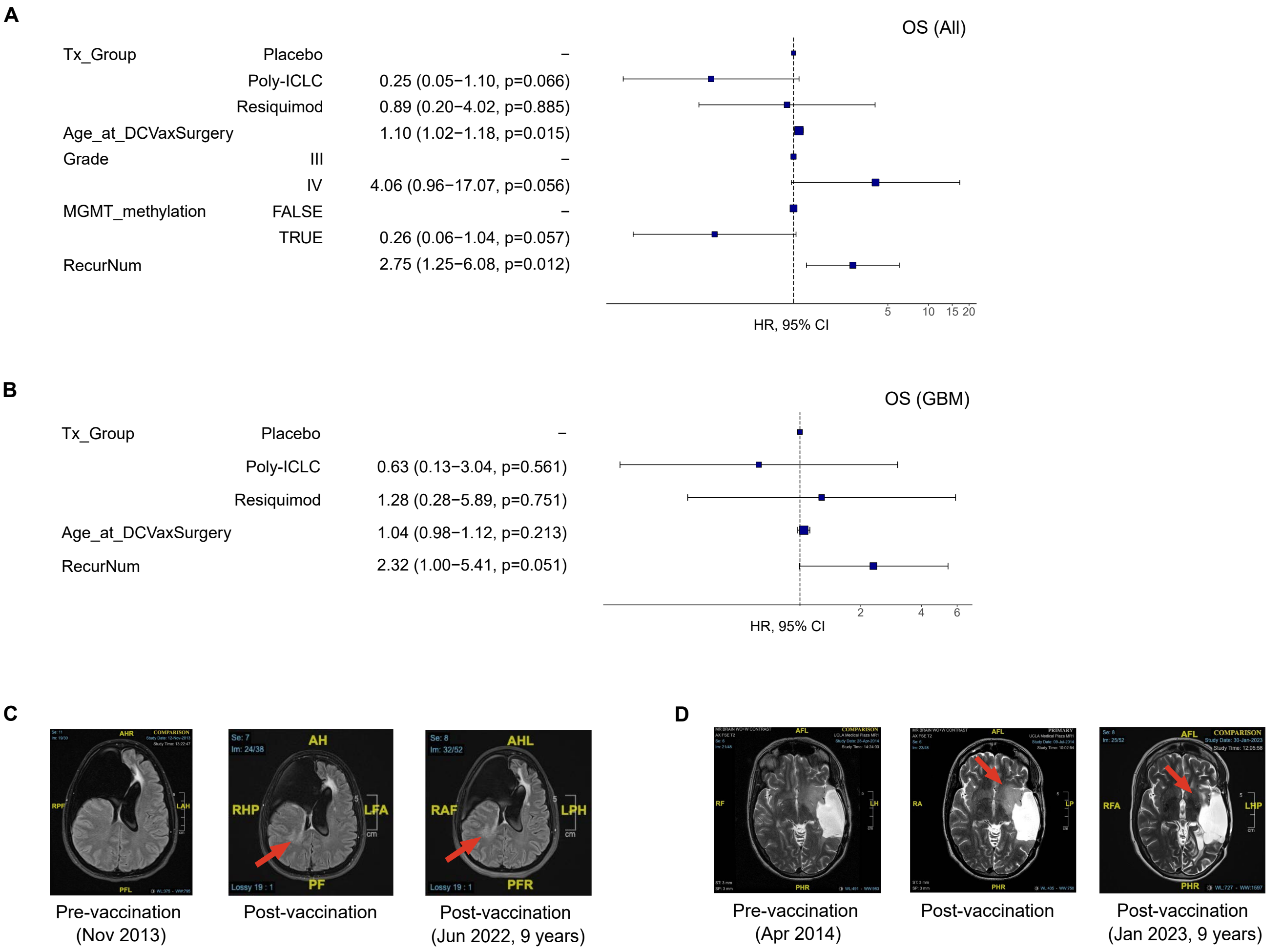

Supplementary Figure 3. The association between combined ATL-DC vaccine and TLR agonist and patient survival.

A, B, Multivariate Cox proportional hazards analysis assessing the hazard ratios of death in TLR agonist treatment groups against placebo in all patients (**A**) or GBM subset (**B**) after adjusting for other clinical covariates (Tx_Group=treatment group, RecurNum=number of recurrences prior to ATL-DC treatment; the CoxPH model did not converge when MGMT_methylation was included). In the forest plot, the squares are the hazard ratio (HR) estimates, the error bars are 95% confidence interval (CI) of the HR, the P value of each covariate is based on its Wald statistics, the P values are not adjusted. In (**A**), the sample distribution in each covariate is Tx_Group: placebo=5, poly-ICLC=9, resiquimod=9; Grade: III=8, IV=15; MGMT_methylation: True=8, False=15. In (**B**), Tx_Group: placebo=4, poly-ICLC=5, resiquimod=6.

C, D, Representative contrast-enhanced MR imaging of patients treated with ATL-DC + poly-ICLC showing initial increase of T2/FLAIR MRI signal (red arrows), which either persists (**C**) or regresses (**D**) over time. Both patients have significantly longer PFS and OS than the rest of the patients in the cohort.

Supplementary Figure 4

A HALLMARK_INTERFERON_GAMMA_RESPONSE (GSVA score, post-Tx, OS)

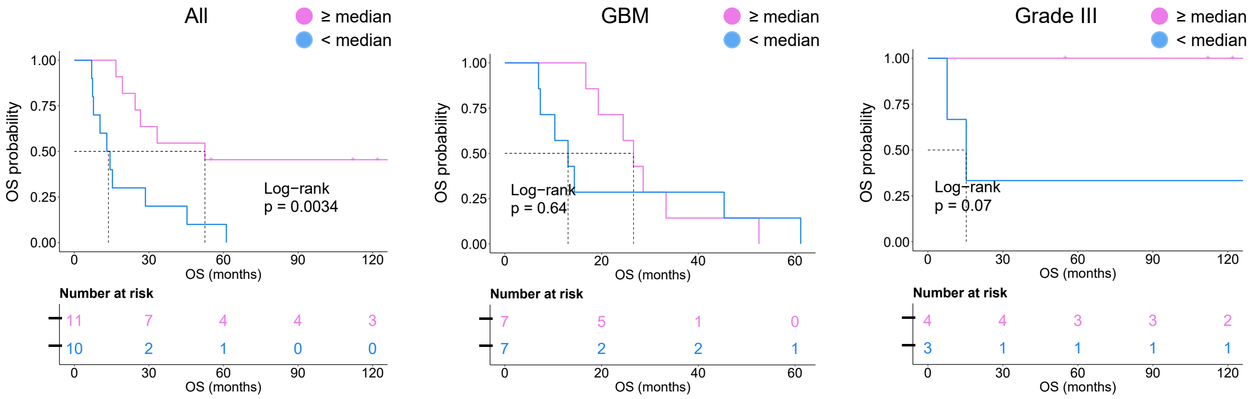

B

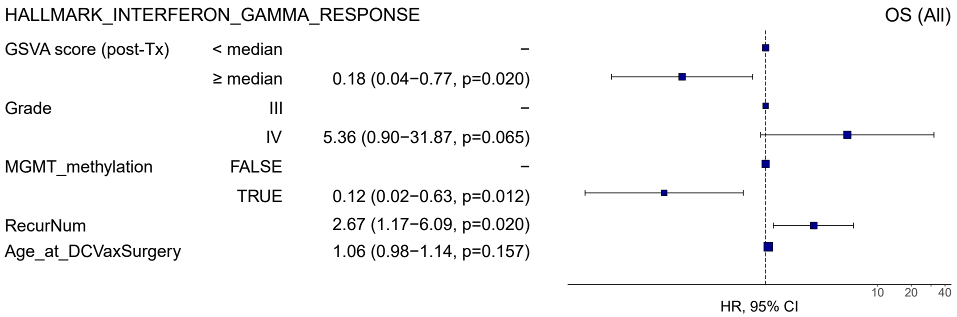

Supplementary Figure 4. The association between IFN pathway activation and overall survival after ATL-DC vaccine and TLR agonist therapy.

A, Kaplan-Meier overall survival curves of all patients (left), GBM (center), and Grade III glioma subsets (right) stratified by their HALLMARK_INTERFERON_GAMMA_RESPONSE GSVA scores in their post-treatment PBMCs. P values, log-rank test.

B, Multivariate Cox proportional hazards analysis assessing hazard ratios of death in patients with high HALLMARK_INTERFERON_GAMMA_RESPONSE GSVA score after adjusting for other clinical covariates. In the forest plot, the squares are the hazard ratio (HR) estimates, the error bars are 95% confidence interval (CI) of the HR, the P value of each covariate is based on its Wald statistics, the P values are not adjusted. T the sample distribution in each covariate is GSVA score (post-Tx): <median=10, ≥median=11; Grade: III=7, IV=14; MGMT_methylation: True=7, False=14.
