# Supplementary material for: TLR agonists polarize interferon responses in conjunction with dendritic cell vaccination in malignant glioma: a randomized phase II Trial
Source: Nat Commun. 2024 May 8;15:3882. doi: 10.1038/s41467-024-48073-y (PMC11078958; doi:10.1038/s41467-024-48073-y)
Supplement: Supplementary file 3 — Description of Additional Supplementary Files [file 41467_2024_48073_MOESM3_ESM.pdf]

Description of Additional Supplementary Files

Supplementary Data 1. Patient level clinical characteristics.

Supplementary Data 2. Patient-level gene expression and gene set scores from bulk RNA-seq data.

A, Difference in log fold changes in ATL-DC+TLR agonist groups compared to ATL-DC+placebo. P values, two-sided Welch t test. P value adjustment, FDR method.

B, Gene sets within ENRICHR database that are significantly overlapping with DEGs upregulated in TLR agonist-treated groups (poly-ICLC or resiquimod, average log2 FC ≥ 1, nominal P-value ≤ 0.05). P values, Fisher's exact test. Q values, FDR-adjusted p values.

C, Patient-level gene expression changes in pre- vs. post-treatment samples (log fold changes). This is the raw data for the gene expression boxplots.

D, Patient-level GSVA gene set score change in pre- vs. post-treatment samples (computed over c2 cgp, c6, c7 and hallmark subsets of MSigDB). This is the raw data for the GSVA score boxplots.

E, Patient-level GSVA score of interferon related gene sets in pre- vs. post-treatment samples. This is the raw data of the GSVA heatmap.

Supplementary Data 3. Cell population identification in CyTOF and single cell RNAseq datasets.

A, CyTOF sample characteristics.

B, CyTOF marker list.

C, Differentially expressed CyTOF markers computed by linear mixed model analysis. P values, unpaired two sided t test. P value adjustment across timepoints, Holm method.

D, Sample-level fractions of cell types identified by unsupervised clustering of the CyTOF dataset.

E, Single cell RNAseq sample characteristics.

F, Cell types identified by cluster-specific, differentially expressed transcripts in the single cell RNAseq dataset. P values, MAST method. P value adjustment, Bonferroni method.

G, Differentially expressed genes in the post-treatment samples of each treatment group (placebo, poly-ICLC and resiquimod) with respect to all pre-treatment samples. This is the raw data of the single cell RNAseq heatmap. P values, MAST method. P value adjustment, Bonferroni method.

Supplementary Data 4. Univariate and multivariate analyses testing the association between IFN gene set scores and patient survival.

A, The association between progression free survival (PFS) or overall survival (OS) and the GSVA scores of select type I and type II interferon gene sets (univariate analysis, P values, log-rank; preTx=GSVA score of pre-treatment sample, postTx=GSVA score of post-treatment sample, diff_preTx_postTx=postTx-preTx).

B, Cox proportional hazard analysis testing the association between PFS or OS and the GSVA scores of select type I and type II interferon gene sets. Concordance, proportion of all pairs of patients whose survival time can be ordered such that the patient with the higher predicted survival is the one who survived longer. P value of the Cox proportional hazard model having zero coefficients, Log-rank test. HR P values are computed based on their Wald statistics. P values shown are not adjusted for multiple comparison.
